# New Advances in Wide-Bandgap RF and Power Electronic Devices: From Material Innovation to System Integration

**DOI:** 10.3390/mi17060673

**Published:** 2026-05-29

**Authors:** Hao Lu, Qinzhu Sun

**Affiliations:** State Key Discipline Laboratory of Wide Band-Gap Semiconductor Technology, School of Microelectronics, Xidian University, Xi’an 710071, China; 23009300262@stu.xidian.edu.cn

## 1. Introduction

With the rapid development of 5G and beyond 5G (B5G) wireless networks, global connectivity is undergoing a revolutionary transformation. The proliferation of cutting-edge applications such as smart cities, autonomous vehicles, and the Internet of Things (IoT) has greatly improved quality of life, while also imposing higher demands on data transmission speed and reliability. At the same time, the continued expansion of the electric vehicle (EV) market is driving the transportation sector toward a more efficient and sustainable future, significantly reducing carbon emissions and reliance on fossil fuels. Together, these technological advances have generated a tremendous demand for high-performance semiconductor devices, especially in the fields of radio frequency (RF) [1,2,3,4,5,6,7,8,9]. Against this backdrop, wide-bandgap (WBG) semiconductor devices (such as gallium nitride GaN, gallium oxide Ga_2_O_3_, aluminum nitride AlN, etc.) play a key role in achieving high-efficiency, high-reliability RF power amplification and power conversion [10], thanks to their excellent breakdown field strength, high electron mobility, and superior high-temperature stability [11,12,13,14,15,16]. This Special Issue aims to bring together the latest frontier achievements in novel RF and power electronic devices and their application areas. This issue includes original research and review papers covering, but not limited to, the following topics: wide-bandgap devices (GaN, Ga_2_O_3_, AlN, etc.) and their applications [17,18,19]; high-frequency RF/millimeter-wave devices [20,21,22,23,24,25] and applications; advanced device processes; device reliability and characterization; semiconductor gate drive design; and WBG-based power converters [26]. This Special Issue not only focuses on innovations in underlying materials (such as ultra-thin barrier layer optimization and novel composite microstructures) but also explores their implementation in specific devices such as high-frequency resonators, high-power amplifiers, surface acoustic wave filters, and display driver circuits, ultimately extending to system-level integration [27] and applications.

As shown in the list, thirteen contributions in this issue are systematically divided into three core sections based on device functions and system applications. In the wide-bandgap power devices section, the included studies cover an article on optimizing dual-channel GaN HEMTs using ultra-thin AlN back barrier layers [Contribution 1], in situ characterization work revealing the electrothermal failure mechanisms of GaN Schottky diodes [Contribution 2], and extreme bandgap AlGaN HEMTs based on quantum well designs [Contribution 3], demonstrating significant improvements in breakdown voltage and reliability through band engineering. In the RF front-end and circuits section [28,29], it includes a compact SPICE model for terahertz detectors [Contribution 4], 24/48 GHz voltage-controlled oscillators based on noise-filtering techniques [Contribution 5], 40 GHz low-noise amplifiers with high image rejection ratios [Contribution 6], and W-band GaN power amplifiers achieving watt-level output [Contribution 7], complemented by a review of low-power all-digital phase-locked loops [Contribution 8] to provide a comprehensive view of frequency synthesis. And in the integrated technology and system applications section, contributions include a bidirectional ESD protection circuit for p-GaN gates [Contribution 9], low-power AMOLED driving circuits based on a-IGZO transistors [Contribution 10], a review of research progress on AlN-based bulk acoustic wave resonators [Contribution 11], miniaturized wideband BAW filters based on high-Q active inductors [Contribution 12], and a W-band through-wall radar system demonstration using a high-gain frequency-scanning antenna [Contribution 13], collectively validating the complete technological chain from material innovation to system integration.

## 2. Structural Innovations and Physical Mechanisms of Wide-Bandgap Power Devices

Al-Mamun and colleagues [Contribution 1] investigated the reliability issues of gallium nitride on sapphire (GaN-on-sapphire) Schottky diodes under extreme conditions, delving into the electrothermal failure mechanisms under combined high temperature and forward bias stress. This study aims to reveal transient degradation processes that are difficult to capture using conventional ex situ analyses, particularly the microscopic evolution of the metal–semiconductor interface under thermo-electrical coupling. The core innovation lies in using a custom-designed microelectromechanical systems (MEMSs) chip to achieve in situ synchronous heating and electrical bias control within a transmission electron microscope (TEM), enabling real-time observation of the entire process from the initiation of microscopic defects to macroscopic catastrophic failure at nanometer resolution. Experimentally, the research team fabricated Schottky diodes with an Au/Pd stack structure and processed them into thin slices for gradient experiments inside the TEM. The results showed that, under lower stress (3 V, 185 °C), the devices could remain stable, but when conditions worsened to 4 V and 300 °C, edge electric field concentration and dislocation networks caused by lattice mismatch jointly accelerated metal atom diffusion, leading to alloying of the Au/Pd layers and their penetration into the GaN layer. Geometric phase analysis (GPA) further confirmed that this electrothermal effect induced significant local lattice strain. When stress reached the critical point (5 V, 455 °C), severe interdiffusion and the formation of intermetallic compounds completely destroyed the integrity of the Schottky barrier, resulting in device failure. Overall, this work, through high-spatiotemporal-resolution in situ characterization, clarifies that defect-mediated electrothermal degradation and interfacial chemical reactions are the key factors leading to the failure of GaN Schottky diodes under high-temperature bias.

Shur et al. [Contribution 2] proposed an extreme bandgap AlGaN high electron mobility transistor (HEMT) based on a quantum channel (QC) design, aiming to overcome the physical limits of conventional GaN-based power devices in breakdown field and gate control capability. This study used metal–organic chemical vapor deposition (MOCVD) technology to grow an Al_0.87_Ga_0.13_N/Al_0.64_Ga_0.36_N/Al_0.87_Ga_0.13_N heterojunction structure on a single-crystal bulk AlN substrate. The two ultra-high aluminum composition barrier layers above and below confine the two-dimensional electron gas (2DEG) within an extremely thin channel. Unlike conventional HEMTs, where the energy band tends to flatten near the threshold voltage, the quantum well band profile of this structure is dominated by the polarization electric field. Even at low sheet densities, it can maintain a strongly confining triangular potential well, thereby raising the ground state energy level above the conduction band minimum, effectively increasing the bandgap width. The experimentally fabricated devices achieved an average breakdown field of up to 11.37 MV/cm with a gate-to-source distance of 0.65 μm, far exceeding the theoretical value calculated from material composition (9.8 MV/cm). The study indicates that this significant improvement arises from a dual mechanism: first, the effective bandgap widening brought by the quantum confinement effect; second, the “quantum real-space transfer” (QRST) of high-energy electrons, where hot electrons tunnel into the wider bandgap Al_0.87_Ga_0.13_N barrier layer under high electric fields, utilizing the barrier material’s inherently high breakdown properties to sustain the voltage. Additionally, the strong confinement increases the bulk electron concentration, enhancing screening against impurity scattering and benefiting mobility under low gate voltage. This quantum channel design, by combining polarization engineering and band structure control, provides a novel technical pathway for fabricating RF and power devices that are resistant to extreme environments and have ultra-high breakdown voltages.

Yu et al. [Contribution 3] proposed a materials engineering solution based on an ultra-thin back barrier layer to address the shortcomings of dual-channel GaN HEMTs in off-state characteristics and gate control capability. The core of this study lies in comparing the suitability of two strongly polar materials—aluminum nitride (AlN) and indium aluminum nitride (InAlN)—as the second channel barrier layer. Transmission electron microscopy observations revealed that the ultra-thin InAlN layer exhibits severe indium composition segregation. This microscopic structural inhomogeneity introduces alloy disorder scattering and interface roughness scattering, resulting in a significant reduction in the carrier mobility of the second channel, thereby degrading the device’s dynamic response and RF performance. In contrast, the AlN layer, due to its excellent crystal quality and high polarization strength, not only effectively blocks gate leakage paths but also significantly enhances the gate’s electrostatic control over the channel. In terms of device performance validation, devices equipped with an AlN back barrier demonstrated comprehensive advantages: their saturated output current density reached 1248 mA/mm, far exceeding the 1150 mA/mm of InAlN devices; more importantly, thanks to the effective shielding of buffer layer traps by the AlN layer, the device breakdown voltage jumped to 294 V, nearly double that of the InAlN device (121 V). In RF applications, due to the lower drain lag ratio of AlN devices, they achieved a power-added efficiency of 57% and an output power density of 11.3 W/mm under 3.6 GHz continuous-wave testing, significantly outperforming the InAlN devices with 41.4% and 8.69 W/mm. This work, through detailed materials characterization and electrical testing comparison, established the role of ultra-thin AlN as an ideal back barrier layer for dual-channel HEMTs, providing important experimental evidence for the design of high-linearity, high-voltage RF power amplifiers.

## 3. High-Frequency RF Front-End and Modeling for 5G/B5G

Liu et al. [Contribution 4] proposed an improved compact SPICE model based on nonlinear transmission line theory to address the insufficient accuracy of terahertz (THz) plasma field-effect transistor (TeraFET) detectors in circuit simulations. This study aims to overcome the limitations of traditional models in handling non-uniform carrier density oscillations and electron inertia effects within the channel, particularly the quantization deviations under resonant detection conditions. The core innovation lies in abandoning the previous simplification of uniformly distributing the Drude inductance, instead adopting an improved segmentation approach that independently calculates the equivalent conductance, capacitance, and inductance for each channel segment based on the node voltage of each segment, thereby accurately capturing the electron inertia effects along the channel. Methodologically, the research team calibrated the DC characteristics using Sentaurus TCAD and embedded the improved model into both the MOSA1 and EKV frameworks, systematically comparing it with analytical theory, COMSOL multiphysics simulations, and experimental measurement data. The results indicate that, under strong resonant modes involving long-channel high-mobility or short-channel low-mobility conditions, this non-uniform model shows significantly enhanced quantitative agreement with benchmark data, effectively correcting the response underestimation problem of traditional models caused by neglecting nonlinear dependencies. Overall, this work introduces non-uniform transmission line parameters, significantly improving the TeraFET simulation prediction capability in the terahertz band and providing a more reliable tool for the design and optimization of related optoelectronic integrated circuits.

Ku et al. [Contribution 5] addressed the challenge in millimeter-wave transceiver systems where voltage-controlled oscillators (VCOs) struggle to balance phase noise and power consumption. Using TSMC 90 nm CMOS technology, they developed two low-noise oscillators operating at 24 GHz and 48 GHz, respectively. The core strategy of this study was based on the principle that nonlinear effects at the tail current source generate second-harmonic noise, which is down-converted near the carrier frequency. Innovatively, an LC filter network resonating at 2f_0_ was paralleled at the tail current mirror to bypass harmful second-harmonic noise to ground, thus greatly suppressing phase noise without significantly increasing power consumption. For the 48 GHz high-frequency design, the team abandoned simply reducing transistor size and instead adopted a push–push architecture combined with a λ/4 slow-wave transmission line. The open-circuit feature of the transmission line at a quarter wavelength served as an RF choke, overcoming the process cutoff frequency limitation and effectively reducing chip area. Regarding measured results, the 24 GHz single-core VCO achieved an excellent phase noise performance of −97.19 dBc/Hz@1MHz with only 6.12 mW power consumption—about an 8 dB improvement compared to the design without the filter, with a tuning range of 6.5%. The push–push 48 GHz VCO successfully output a 49.8 GHz frequency-doubled signal, with a phase noise of −80.52 dBc/Hz@1MHz at 6.89 mW power and a tuning range of 7.2%. Although the 48 GHz output power was slightly lower due to frequency doubling loss, the core area was controlled within 0.5 mm^2^, and it exhibited a higher figure of merit (FOM) compared to similar works. This work demonstrates the feasibility of achieving high-performance millimeter-wave local oscillators on mature process nodes through a combination of noise-filtering technology and transmission line engineering, making it highly suitable for short-range radar and 5G/6G RF front-end modules.

Guo et al. [Contribution 6] proposed a high-suppression-ratio low-noise amplifier (LNA) design based on a switchable transformer notch filter to address the problem of signal-to-noise ratio degradation caused by image frequency interference in 5G millimeter-wave receivers. The core of this study lies in integrating a transformer with a switched capacitor array in the inter-stage matching network and using transmission zero-pole control technology to simultaneously achieve impedance transformation and image suppression without increasing additional chip area. The article deeply analyzes the variation in pole-zero distribution when the port impedance changes from real to complex, indicating that, in a complex impedance environment, the secondary pole positions basically remain unchanged, while only the primary poles shift with the capacitance values. This characteristic provides a theoretical basis for precise frequency tuning. For the circuit implementation, the team designed a two-stage differential neutralized common-source structure based on a 65 nm CMOS process. By optimizing the transformer coupling coefficient (k = 0.13) and the coil quality factor (Q ≥ 16 at 40 GHz), the notch network ensures a high-Q transmission zero at 28 GHz. Experimental results show that the LNA achieves a peak gain of 18 dB and a noise figure of 4.4 dB in the 36.3–40.7 GHz band, while the image rejection ratio (IRR) is increased to 53.4 dB at an intermediate frequency of 6 GHz, and the IRR remains above 40 dB across the entire operating band. By adjusting the switched capacitor states, the circuit maintains high linearity (IP1 dB ≈ −16 dBm) with a core area of only 0.13 mm^2^ and a power consumption of 25.4 mW. It achieves high image suppression performance comparable to advanced technology nodes through coordinated pole-zero design on a mature process node, providing a compact and efficient solution for millimeter-wave multi-band transceivers.

Liu et al. [Contribution 7] addressed the bottleneck of insufficient output power from solid-state power sources in W-band (75–110 GHz) wireless communication and radar systems. Based on 130 nm AlGaN/GaN-on-SiC high electron mobility transistor (HEMT) technology, they designed and realized a balanced monolithic microwave integrated circuit (MMIC) power amplifier with both high power and high efficiency. The core strategy of this study was to use the reflection cancelation characteristics of the balanced architecture to improve input and output matching, while employing a three-stage amplification structure with an asymmetric gate width ratio of 1:2:4, gradually enhancing the drive capability to support high-power output at the final stage while ensuring a linear gain of 17 dB. To address the problem of excessive losses and processing difficulties of lumped components in the millimeter-wave frequency band, all stages and input/output matching were implemented using high- and low-impedance microstrip lines, and a quarter-wavelength high-impedance line was used to construct the bias network, effectively avoiding parasitic oscillations. In terms of device technology, relying on the excellent thermal performance of the 4H-SiC substrate and the high breakdown characteristics (>40 V) of the GaN heterojunction, the chip exhibited outstanding performance in the 80–86 GHz band. Small-signal tests showed a gain exceeding 17 dB; under large-signal pulse test conditions (drain voltage 18 V, gate voltage −2.2 V), the saturated output power exceeded 35 dBm (approximately 3.2 W) at an input power of 22 dBm, with a peak power-added efficiency (PAE) of up to 24% and an average efficiency remaining above 22%. Compared with the existing literature, this work not only achieved watt-level output but also maintained efficiency metrics at the forefront of current W-band GaN power amplifiers, with a chip size of only 2.65 mm × 3.75 mm. This study fully exploited the potential of GaN technology in the millimeter-wave band through optimized topology and transmission line design, providing a strong transmitter solution for next-generation high-resolution imaging radar and high-speed backhaul systems.

Navaneethan et al. [Contribution 8] systematically reviewed the latest architectural evolution and technical bottlenecks of microwatt-level all-digital phase-locked loops (ADPLLs) in response to the urgent demand for ultra-low-power RF front-ends in the Internet of Things (IoT) and wearable devices. The review points out that, as semiconductor processes enter deep sub-nanometer nodes, traditional analog charge-pump PLLs, relying on large off-chip loop filters and struggling to cope with PVT variations, are gradually being replaced by digital architectures. The article focuses particularly on divider-less architectures, which are considered key to breaking the milliwatt-level power barrier because they completely eliminate the N^2^ noise multiplication effect and dynamic power consumption caused by multi-mode dividers (MMDs). The authors categorize existing low-power ADPLLs into four types: accumulator-based, sub-sampling (SS-PLL), injection-locked, and hybrid, and thoroughly analyze the trade-offs in phase noise, locking time, and circuit complexity for each type. In technical detail, the review elaborates on how to reduce the quantization range through digital time converter (DTC)-assisted time-to-digital converters (TDCs), thereby lowering TDC sampling rate and power consumption—for example, by using a DTC to pre-align the reference clock, allowing the TDC to handle only a small residual phase error. Additionally, the article explores innovative digitally controlled oscillator (DCO) designs that use transformer coupling to improve the resonator’s quality factor (Q-factor), such as inverse Class-F (Class-F^−1^) oscillators and resonant stacked-gm structures, which significantly enhance oscillation amplitude and phase noise performance without increasing current consumption. By comparing over twenty leading works (covering 65 nm to 7 nm processes), the review clearly identifies that switchable dual-loop hybrid architectures combining divider-assisted and sub-sampling paths, as well as accumulator designs with embedded TDCs, represent the most promising approach to achieving sub-300 μW power consumption while maintaining excellent jitter performance, providing a comprehensive technical roadmap for designing frequency synthesizers in future energy-harvesting wireless sensor nodes.

## 4. High-Reliability ESD Protection, Display, Filter, and System Applications

Zhang et al. [Contribution 9] proposed a novel protection circuit architecture with bidirectional clamping function to address the shortcomings of p-GaN gate high electron mobility transistors (HEMTs) in the field of electrostatic discharge (ESD) protection. This study aims to solve the challenge that traditional silicon-based or single-diode protection schemes struggle to balance bidirectional protection capability with low static power consumption, particularly tackling the critical issue that the p-GaN device gate is prone to breakdown due to transient overvoltage. The core innovation lies in designing two topologies (Clamp1 and Clamp2) based on diode strings working collaboratively with p-GaN HEMTs, using HEMTs as controlled switches that rapidly turn on to discharge–charge when detecting an ESD pulse, thereby clamping the gate-source voltage within a safe threshold. Methodologically, the team combined theoretical derivation with PCB-level experiments, focusing on analyzing the variation law of trigger voltage (V_Tri_) under static and transient conditions. The study found that the first-generation design (Clamp1), while capable of withstanding high secondary breakdown current, had an excessively low clamping voltage under static conditions, making false triggering unavoidable; the improved Clamp2 design introduced a current-limiting resistor to construct a voltage divider network, successfully achieving high impedance under static conditions and precise triggering under transient conditions. While maintaining nanoampere (nA)-level ultra-low leakage current, it passed voltage stress tests exceeding 2 kV under the Human Body Model (HBM). Experimental data indicate that, by adjusting the value of the current-limiting resistor, the forward trigger voltage can be precisely controlled within the 5 V to 8 V safe window, with almost no impact on the main power transistor’s switching characteristics. All in all, his work demonstrates an integrated solution that not only meets industrial-grade ESD robustness standards but also significantly reduces system additional power consumption, providing an important reference for the monolithic integrated protection design of GaN power chips.

Chang et al. [Contribution 10] proposed a low-power circuit design scheme based on n-type amorphous indium gallium zinc oxide (a-IGZO) transistors to address the pain point in AMOLED display panels where the energy consumption of the emission pulse (EM) driving circuit increases sharply with pulse width. The core of this study lies in reconstructing the internal topology of the inverter module, completely eliminating the inherent direct current path in traditional single-type transistor inverters, making the circuit’s power consumption no longer constrained by pulse duration. To address the leakage current issue commonly caused by negative threshold voltage (depletion mode) in a-IGZO processes, the team introduced dual low-voltage power rails and used a series dual transistor (STT) structure as a physical isolation barrier. In circuit implementation, the design uses only 12 transistors and 2 capacitors, achieving precise control of the QB node through optimized clock phase alignment. SPICE simulations based on a 6.1-inch UHD display panel show that, at a refresh rate of 120 Hz, the total circuit power consumption can be stably maintained between 0.568 mW and 0.836 mW, regardless of whether the pulse width is as short as 3 lines or as long as 2157 lines, and it exhibits strong robustness across a wide threshold voltage drift range of −4.0 V to 2.5 V, achieving significantly improved energy efficiency compared to traditional diode-load solutions. This work provides a practical design approach for future high-resolution, low-power display backplane technology through clever timing control and circuit architecture innovation.

Lu et al. [Contribution 11] systematically reviewed the latest research progress of aluminum nitride (AlN)-based bulk acoustic wave (BAW) resonators in response to the urgent demands of 5G communications for high frequency, wide bandwidth, and high-power handling in RF front-end filters. The review points out that, as the core component of BAW filters, AlN thin films dominate due to their excellent chemical stability, high thermal conductivity, and CMOS process compatibility; however, the low piezoelectric coupling coefficient of pure AlN limits its further application in wideband systems. The article discusses in detail strategies to enhance the electromechanical coupling coefficient (k_eff_^2^) by doping with scandium (S_c_) to form ScAlN alloys, while analyzing challenges such as the decrease in quality factor (Q) and deterioration of temperature coefficient (TCF) caused by doping, and introduces hybrid structures using SiO_2_ layers for temperature drift compensation. In terms of device architectures, the study compares thin-film bulk acoustic resonators (FBARs) and solidly mounted resonators (SMRs) in terms of structural strength, heat dissipation paths, and performance, particularly highlighting the potential of new technologies like Xtended bulk acoustic wave (XBAW) for bandwidth expansion. Additionally, the review covers frequency-reconfigurable filters, improvements in ladder and lattice circuit designs, as well as design optimization algorithms. Overall, this work integrates the latest developments in material epitaxial growth (such as the two-step MOCVD and PVD methods), structural innovations, and design optimizations, providing a comprehensive technical reference and theoretical support for the development of high-performance AlN-based BAW filters in the future.

Xu et al. [Contribution 12] proposed a miniaturized wideband filtering solution based on high-Q active inductors to address the dual challenges faced by bulk acoustic wave (BAW) filters in radio frequency front-ends during the 5G/6G evolution: limited bandwidth and excessive area occupied by passive inductors. Traditional BAW filters, in order to cover the 2 GHz bandwidth required for Wi-Fi 6E/7, typically need to integrate between three and four off-chip passive inductors, which not only makes it difficult to shrink the package size, but also causes significant decreases in quality factor (Q) due to mutual inductance effects between inductors. To address this bottleneck, the study designed a novel three-stage NMOS active inductor topology, which effectively suppresses the input parasitic capacitance by introducing a miniaturized intermediate transistor stage and compensates for gain using a two-stage amplification structure, thereby achieving a peak Q of up to 4000 and a tunable inductance range of 1 to 10 nH in the 2–7 GHz frequency range. At the system integration level, the team abandoned the traditional ladder-type bandpass architecture and instead adopted a reconfigured topology based on the band-stop principle, embedding the active inductor into the series resonance arm to generate transmission zeros. Joint simulations using ADS and Cadence show that, compared with a passive inductor solution with a Q of 50, this hybrid filter not only reduces insertion loss to −1.1 dB in the 4.55–5.05 GHz band but also improves out-of-band suppression on both sides to −35 dB and −53 dB, respectively. More importantly, the area of a single active inductor is only 30 × 30 μm^2^, reducing the overall filter chip size to 0.83 × 0.75 mm^2^, providing a highly competitive solution for next-generation highly integrated RF modules.

Tian et al. [Contribution 13] developed a high-gain frequency-scanning antenna based on a sinusoidally modulated reactance surface (SMRS) to address the challenges of system complexity and high cost faced by through-wall radar (TWR) in W-band applications. For traditional W-band radar, if a phased array solution is used, beam control relies on complex phase shifters and fabrication is extremely difficult; if mechanical scanning is used, there are drawbacks such as wear-prone rotating parts and slow speed. To solve this issue, the study utilized the leakage wave radiation principle of spoof surface plasmon polaritons (SSPPs) and designed quasi-H-shaped periodic metallic units. By controlling the unit height to modulate the propagation constant of surface waves, beam scanning is achieved purely through frequency control without the need for phase shifters. Structurally, the antenna employs full-metal CNC machining processes, completely eliminating the dielectric substrate, fundamentally avoiding the high dielectric loss and power handling bottlenecks in the millimeter-wave band. To compensate for the generally low gain of leakage wave antennas, the team integrated a horn-shaped expansion structure at the radiation aperture, significantly enhancing energy concentration capability, with measured gain increasing by about 10 dB compared to the non-horn structure, reaching a peak of 20.9 dB. Experimental results show that, within the 92.8–97.6 GHz operating band, the antenna has a reflection coefficient better than −10 dB, a scanning rate of up to 4.05°/%, and sidelobe suppression below −10 dB. To verify practicality, the researchers integrated it into a vehicle-mounted W-band radar system. Field tests demonstrated that, whether stationary or moving at 13 cm/s, the system could penetrate a 20 cm thick wooden wall from a distance of 10 m and clearly distinguish multiple corner reflector targets behind the wall, with ranging error controlled within 5 cm. This work, through an all-metal SMRS-SSPP design, achieves high-performance beam scanning with a simplified single-channel feed structure, providing a low-cost, highly reliable through-wall detection solution for urban counter-terrorism, disaster rescue, and other scenarios.

We hope that this Special Issue on antennas and filters will offer readers a good overview of the current state-of-the-art developments in these fast-growing areas of research as well as an introduction to some of the newest techniques developed in this field.
